# Discerning Unmet Self-Care Need and Co-Design of a Mobile App With Chinese Immigrant Caregivers to Promote Self-Care

**DOI:** 10.1093/geroni/igaa057.088

**Published:** 2020-12-16

**Authors:** Kexin Yu, Haojun Jiang, Mandong Liu, Shinyi Wu, Maryalice Jordan-Marsh, Iris Chi

**Affiliations:** 1 University of Southern California, Palo Alto, California, United States; 2 Nanjing Agricultural University, Nanjing, China; 3 University of Southern California, Los Angeles, California, United States

## Abstract

Immigrant caregivers are the backbone for supporting frail older adults in the United States. However, caregivers' own self-care need is often neglected, especially among racial/ethnic minorities. In the current study, a co-design approach was employed to develop a mobile app for raising awareness and promoting behaviors of self-care among Chinese immigrant caregivers. Individual in-depth interviews conducted through two co-design phases, i.e., conceptual design and prototype design. Twelve caregiver participants voiced their unmet self-care need and expressed their opinions about adopting the co-designed mobile App to access self-care information. The unmet self-care need includes both self-care barriers and supportive resources, and App features the caregivers wish to have. Three self-care barriers were identified in the co-design interviews, including culture-specific stressors, immigrant-status related challenges, and work-related restrictions. Caregiver participants expressed that they wish the care recipients could have education on the boundaries between them and a caregiver. Additionally, the participants reported wanted to learn about handling care emergencies with the co-design app and would like to see short videos and stories included in the App. Participants expressed mixed opinions towards adopting mobile technology – while most of the participants appreciated the content provided, some were concerned that learning with mobile technology could add to their burdens, and technical difficulties could prevent them from using the App. Designing the App to be engaging and fun emerged as highly desirable. The co-design process appears to be beneficial in having participants to articulate both current self-care barriers and preference for a mobile technology tool.

